# Supplementary material for: Global health security and universal health coverage: Understanding convergences and divergences for a synergistic response
Source: PLoS One. 2020 Dec 30;15(12):e0244555. doi: 10.1371/journal.pone.0244555 (PMC7773202; doi:10.1371/journal.pone.0244555)
Supplement: Attachment — Submitted filename: COMMENTS.docx [file pone.0244555.s001.docx]

This is a relevant ecological study that involved 183 countries. The study assessed the relationship between GHS and UHC by examining their association using two recent quantitative indices. However, this study should undergo a major review before considering it for publication.

(1) The authors should replace “association” with “relationship”
(2) It was not clear if the authors covered the following areas (First, we will describe the concordance and discordance of the indicators for UHCI and GHSI. Second, we will analyze the level and equity of UHCI and GHSI across the six WHO regions. Third, we will assess the association between UHCI and GHSI) in the statistical analysis section.
(3) The authors should consider applying control-chart to explain the outliers and countries above and below the median GHS index and UHC index for maternal and child health and infectious diseases. Deploying this method will enable the authors to identify special- and common-cause variation.
(4) It will be more relevant if the authors can examine what made some countries to have discordant associations between universal health coverage index and global health security index.
(5) How did the authors manage missing data?
(6) This is an ecological study that used aggregate data at country-level. Thus I will advise the authors to highlight other important weaknesses of this study.
