# Supplementary material for: Global health security and universal health coverage: Understanding convergences and divergences for a synergistic response
Source: PLoS One. 2020 Dec 30;15(12):e0244555. doi: 10.1371/journal.pone.0244555 (PMC7773202; doi:10.1371/journal.pone.0244555)
Supplement: Attachment — Submitted filename: Rebuttal Letter GHS and UHC PONE November 27 2020.docx [file pone.0244555.s003.docx]

**PONE-D-20-11383R1: Global Health Security and Universal Health Coverage: Understanding Convergences and Divergences for a Synergistic Response**

Dear Dr Irene,

Thank you for giving us the opportunity to improve our manuscript. The comments from the reviewer are very useful and constructive.

We are pleased to submit the third version of our manuscript to PLOS ONE. Following is our point-by-point response to the comments from the reviewers. The comments from the reviewers are shown in Calibri and *Italics*; our responses are in Calibri, Normal and **Bold.**

Kind regards,

Dr Yibeltal Assefa

Corresponding author

**Point-by-point response to the comments**

*Review Comments to the Author*

*Reviewer #2: Thank you for addressing the comments raised earlier about your manuscript. The manuscript is more clearer now. However, the manuscript could be further improved by addressing the following comments:*

*1. Include the p-values for all correlation coefficients in the results section to enable readers assess the significance of the associations.*

**We accept the comment. Thank you. The p-values for all correlation coefficients are provided included in the results section.**

*2. You state that the primary health approach is useful in strengthening health systems. It will be useful to remind readers what the basic idea of PHC is and how it relates to strengthening health systems.*

**We accept the comment. Thank you. We have provided the basic idea and pillars of PHC as follows: The PHC approach facilitates the provision of a continuous and comprehensive care closer to the community by addressing both the supply and demand sides of health care. This will be possible only if there is a political commitment to enhance the three pillars of PHC: universal access to quality health services, empowered people and communities, and multisectoral policy and action for health.**

*3. Under strengths and limitations, you still indicate this …”(3) it uses different analytical approaches (regression analysis and chi-squared statistics) to triangulate the findings of the study;…”. Please remove this statement as you did not perform this analysis.*

**We accept the comment. Thank you. We have removed the statement.**

*4. Under the author summary box :What did the researchers do and find? • “We determined the relationship between GHS and UHC by using two recent quantitative indices: UHC index (UHCI) [1, 2] 1,2 and GHS index (GHSI). We conducted correlation and regression analyses”. Please remove the regression analysis as this was not done. Also include the p-values for the results section in the box as you indicate some significant correlations exist.*

**We accept the comment. Thank you. We have removed regression analysis. The p-values for all correlation coefficients are provided.**

*5. Add p-values to the correlation coefficients in the abstract.*

**We accept the comment. Thank you. The p-values for all correlation coefficients are provided.**

*6. In the conclusion you state “…hence, the tension between GHS and UHC should be transformed into a synergistic planning, financing and implementation, through a diagonal investment and service delivery approach”. Explain the term service delivery approach.*

**We accept the comment. Thank you. We have revised it as follows: service delivery approach, including differentiated, integrated and community-led services.**
